# Mechanism of suppressors of cytokine signaling 1 inhibition of epithelial-mesenchymal transition signaling through ROS regulation in colon cancer cells: suppression of Src leading to thioredoxin up-regulation

**DOI:** 10.18632/oncotarget.11537

**Published:** 2016-08-23

**Authors:** Sung-Hoon Jung, Su-Min Kim, Choong-Eun Lee

**Affiliations:** ^1^ Department of Biological Science, College of Science, Sungkyunkwan University, Suwon 440-746, Korea

**Keywords:** suppressors of cytokine signaling, reactive oxygen species, Src, thioredoxin, EMT signaling

## Abstract

Reactive oxygen species (ROS) participate in malignant progression of cancers including epithelial-mesenchymal transition (EMT). We have investigated the role of suppressors of cytokine signaling (SOCS)1 as an inhibitor of ROS-induced EMT using colon cancer cell lines transduced with SOCS1 and shSOCS1. Hydrogen peroxide treatment induced EMT features such as elevation of vimentin and Snail with a corresponding reduction of E-cadherin. The EMT markers are significantly decreased upon SOCS1 over-expression while increased under SOCS1 knock-down. SOCS1 inhibited ROS signaling pathways associated with EMT such as Src, Jak, and p65. Of note, strong up-regulation of Src activity in SOCS1-ablated cells was responsible for the elevated signaling leading to EMT, as shSrc or Src inhibitor abolished the shSOCS1-induced promotion of EMT response. Suppression of ROS-inducible EMT markers and invasion in SOCS1 over-expressing cells correlated with significantly low intracellular ROS levels in these cells. Analysis of antioxidant enzymes in SOCS1-transduced cells revealed a selective up-regulation of thioredoxin (Trx1), while thioredoxin ablation restored ROS levels and the associated EMT markers. As a mechanism of thioredoxin up-regulation by SOCS1, inhibition of Src activity promoting nuclear translocation of Nrf-2 is proposed. Taken together, our data strongly indicate that SOCS1 antagonizes EMT by suppressing Src activity, leading to thioredoxin expression and down-regulation of ROS levels in colon cancer cells.

## INTRODUCTION

Reactive oxygen species (ROS) have been implicated during initiation, promotion, and malignant progression for cancers of the breast, liver, and colon [1–3]. ROS is also shown to participate in epithelial - mesenchyme transition (EMT) [4–5]. EMT causes epithelial cells to lose their polarity and intercellular adhesion, and then to gain fibroblast-like and invasive properties to become cells with mesenchymal features [6, 7]. Transcription factors such as Snail, Twist, and SIP1 activate EMT by regulating expression of genes involved in cell adhesion and migration [8–10]. EMT is thus considered critical for tumor cell invasion to acquire malignant phenotype [11].

It has been reported that cancer cells under hypoxia exhibited increased intracellular ROS levels and finally progressed to EMT, the process of which was blocked by an anti-oxidant N-acetyl cysteine (NAC) [12]. Both MMP3 and MMP9 are implicated as inducers of ROS leading to EMT in breast and colon cancer cells [13, 14]. In addition, NADPH oxidase (Nox) 1-induced ROS generation appears responsible for EMT and invasion of melanoma cells [15]. While these findings provide insights for the mechanism of ROS generation and ROS-induced EMT process in diverse tumor cells, strategies targeting ROS signal pathways to prevent EMT have not been extensively investigated.

Proto-oncogene Src (c-Src) has been widely reported for transformation, proliferation and metastatic process of diverse tumors [16–18]. Src is shown to induce cell motility and invasion through p38/Akt pathways in breast cancers and glioma [16]. For colon cancer models, deregulation of Csk, a negative regulator of Src induced invasion [17], and the Src signaling pathway was identified as the target of the metastatic suppressor NDGR1 [18]. Through its ability to activate Nox 1 in Rac-dependent mechanism, Src may contribute to the ROS-induced EMT process and invasion of colon cancer cells [19].

Suppressors of cytokine signaling (SOCS), initially found as inhibitors of JAK/STAT pathways, have emerged as multi-functional proteins governing cell growth and differentiation [20, 21]. The tumor suppressive function of SOCS has been demonstrated through the blockade of growth signaling pathways which utilize both receptor and non-receptor tyrosine kinases including EGFR, Mek, Jak, and Src family proteins [22–25]. In breast and hepatic carcinoma cells, expression levels of SOCS1 are down-regulated by the promoter DNA methylation, the reversal of which resulted in the induction of SOCS1 and anti-growth properties [26, 27]. In addition, SOCS may exert tumor suppressive effects by promoting the action of p53 and p21. SOCS1 is shown to enhance p53 activation through binding with p53 [28], while SOCS3 up-regulates p21 expression and cell cycle arrest upon DNA damage [29]. On the other hand, several studies suggested pro-tumor functions of SOCS1 [30, 31]. Upon the analysis of gene expression data sets of human colorectal cancer specimens, SOCS1 expression is found elevated in tumors than normal epithelium. In line with this observation, the over-expression of SOCS1 promoted cell growth and resistance to death stimuli of colon cancer cells [30].

In fact, both pro-apoptotic and anti-apoptotic functions of SOCS1 have been noted depending on the tumor cell types and apoptotic triggers. For example, it has been reported that SOCS1 promoted Fas-induced apoptosis by NF-κB down-regulation [32] whereas it inhibited TNF-α-induced apoptosis by suppression of Jaks in Jurkat T cells [33]. We have observed that during the ROS-mediated apoptosis of immune cells SOCS1 expression was induced by ROS, and SOCS1 over-expression led to the inhibition of oxidant-induced apoptosis. As a mechanism to inhibit ROS signaling, SOCS1 protected protein tyrosine phosphatases (PTP) from inactivation by ROS attack [33].

Based on such anti-oxidant effect of SOCS1 we have hypothesized that SOCS1 may prevent ROS-induced EMT and invasive property of cancer cells, and sought to investigate molecular mechanisms involved. Employing colon cancer cell lines transduced with SOCS1 or shSOCS1, we have obtained data indicating that SOCS1 suppresses intracellular ROS levels and EMT signaling through the inhibition of Src activity. The Src inhibition by SOCS1 led to up-regulation of nuclear Nrf-2 and expression of thioredoxin (Trx1), a key anti-oxidant defense enzyme to down-regulate intracellular ROS. Reduced ROS levels and attenuated EMT signal caused by Src suppression in SOCS1-transduced cells were recovered by thioredoxin ablation, suggesting that thioredoxin and Src form a novel regulatory loop for the anti-EMT function of SOCS1 through ROS.

## RESULTS

### Induction of SOCS1 and changes in EMT markers by hydrogen peroxide

**Figure 1:**
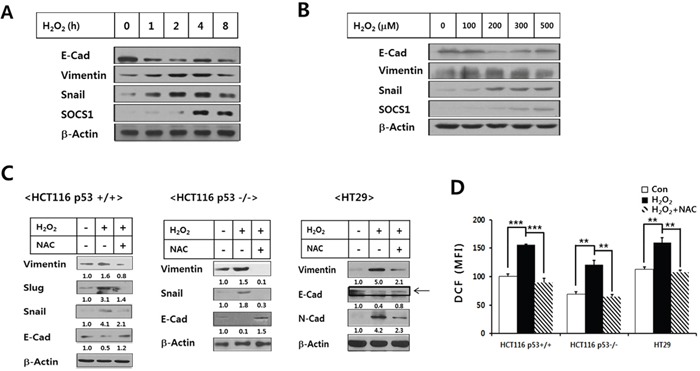
Regulation of EMT markers and SOCS1 expression by H_2_O_2_ and effects of NAC on H_2_O_2_-induced ROS and EMT markers in colon cancer cell lines HCT116 p53 +/+ cells were treated with H_2_O_2_ at 200 μM for different durations **A.**, and for 2 h at increasing doses **B.**, which were then subjected to the immunoblot analysis for SOCS1 and EMT markers. HCT116 p53+/+, HCT116 p53−/−, and HT29(p53mt) colon cancer cell lines were treated with H_2_O_2_ (200 μM, 2 h) with or without pretreatment of NAC for 1 h at 10 mM, after which EMT marker expressions were analyzed **C.** The ROS levels in these cells were determined at 30 min post H_2_O_2_ treatment **D.**

Since ROS signaling is important in the induction of the malignant growth of tumors through EMT, the ROS inhibiting action of SOCS1 to suppress EMT has been a subject of interest. In order to investigate the role of SOCS1 in tumor cell response to ROS, induction of SOCS1 expression by hydrogen peroxide, a direct source of ROS, was first examined. In p53+/+ HCT116 colon cancer cell lines, SOCS1 is induced time- and dose-dependent manners upon H_2_O_2_ stimulation, suggesting a potential regulatory role of SOCS1 during the cellular response to ROS in these cells (Figure 1A and 1B). Under the conditions applied for SOCS1 induction, significant changes in expression levels of EMT markers were observed. Up-regulation of mesenchymal markers, vimentin and Snail with simultaneous down-regulation of epithelial marker E-cadherin was obtained at 200 uM H_2_O_2_ treatment for 2 h (Figure 1A). These conditions were thus considered optimal for the induction of EMT and used for the experiments followed for the parental HCT116 cells.

The EMT marker induction by H_2_O_2_ was observed in the anti-oxidant NAC-sensitive manner both in HCT116(p53 wt) and HCT116(p53 null) as well as HT29(p53 mt) colon cancer cells, indicating that EMT induction by ROS occurred independent of p53 activity in colon cancer cell lines (Figure 1C). Elevation of cellular ROS levels by H_2_O_2_ treatment and its abrogation upon pre-incubation with NAC also exhibited similar patterns in different colon cancer cells, which supported the above notion (Figure 1D). In p53+/+ HCT116 cells, SOCS1 induction was evident from 4 h, kinetically following the maximal induction of EMT markers seen at 2 h (Figure 1A). Likewise, higher levels of SOCS1 induced upon 300 ~ 500 μM H_2_O_2_ treatment were seen with a slightly reduced EMT response as compared with the maximal EMT at 200 μM H_2_O_2_ (Figure 1B). This suggests an anti-EMT function of SOCS1 as a part of the anti-oxidant response.

### ROS-induced EMT signaling and anti-EMT effect of SOCS1 through Src regulation

**Figure 2:**
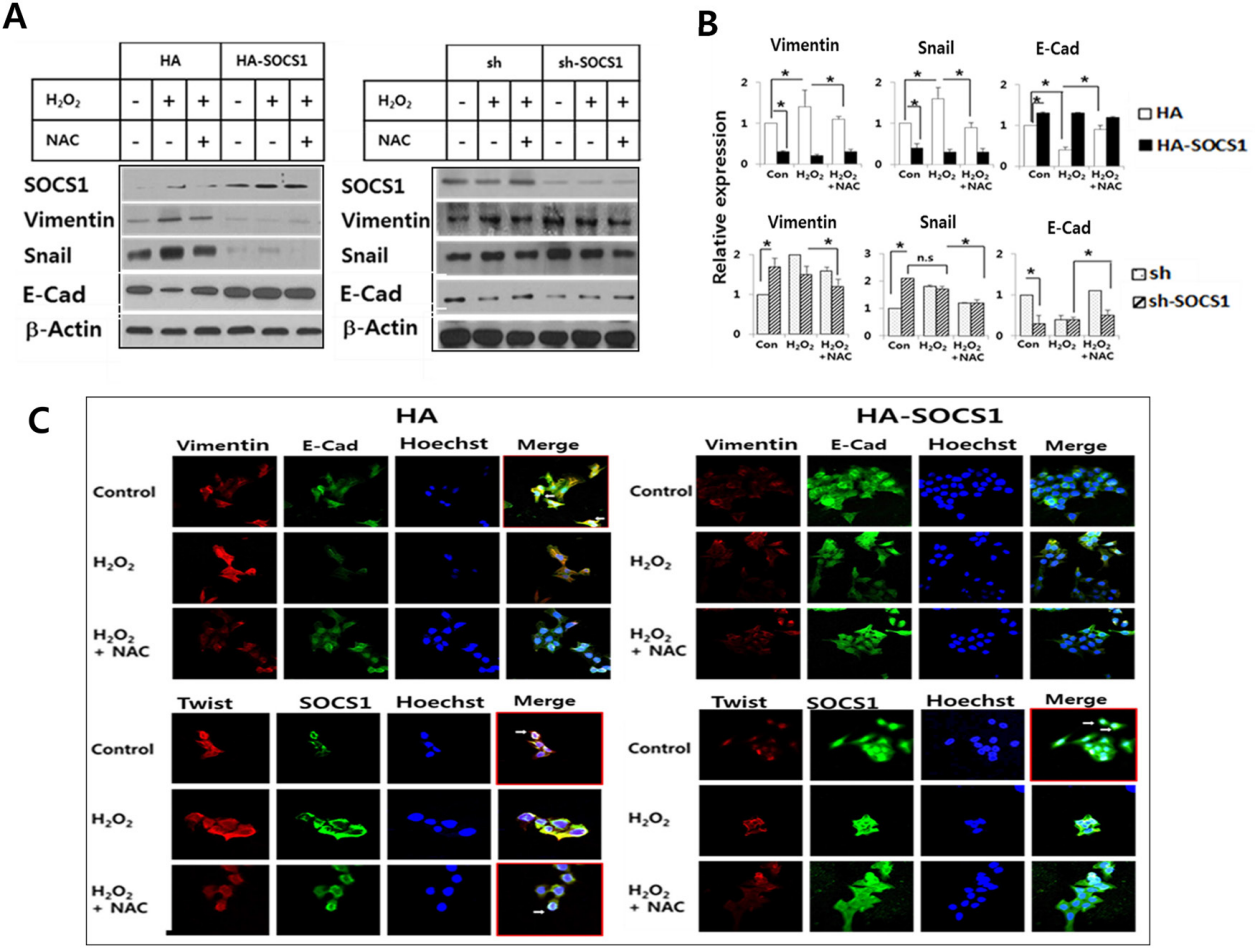
Anti-EMT function of SOCS1: EMT markers were down-regulated in cells transduced with SOCS1, while promoted upon SOCS1 knock-down HA & HA-SOCS1, and sh & sh-SOCS1 HCT116 p53+/+ cells were established by transfection of respective vectors by electroporation as described in Materials and Methods. Cells maintained in the selection media were treated with H_2_O_2_ (300 μM for 2 h) and changes in EMT markers were analyzed by immunoblotting. A representative blot is shown for each cell system **A.** Densitometric analysis of immunoblots normalized to beta-actin showing relative expression of EMT regulators. Data represent mean+SE obtained from 3 independent experiments **B.** HCT116 p53 +/+ HA & HA-SOCS1 cells were treated with H_2_O_2_ as above and then changes in EMT markers and SOCS1 location were examined by immunofluorescence analysis **C.** Vimentin and E-cadherin colocalize at the cell membrane in control HA cells shown in the merged figure (white arrows in upper left panel). SOCS1 colocalizes with Twist in the perinuclear region (Lower left panel). In SOCS1-transduced cells E-cadherin expression levels increased over vimentin (Upper right panel), and SOCS1 expression was seen both in the cytosol and nucleus with reduced Twist expression (Lower right panel).

To investigate the role of SOCS1 in the regulation of ROS-induced EMT, HCT116/p53 wt cells stably over-expressing or knock-down for SOCS1 were established using transduction of HA-SOCS1 or sh-SOCS1 genes as described [33]. As shown in immunoblots and densitometric analysis data (Figure 2A and 2B) the mock cells responded in a manner similar to the parental cell line upon treatment with H_2_O_2_ with induction of vimentin and Snail with decrease in E-cadherin (vimentin and Snail induction by 1.5 to 2.0 fold; E-cadherin reduction by 2 to 3 fold). SOCS1-transduced cells exhibited low levels of EMT markers independent of H_2_O_2_ or NAC treatment. On the contrary, sh-SOCS1 cells were found to have elevated EMT markers. Confocal analysis confirmed the increased expression of vimentin and reduced expression of E-cadherin by H_2_O_2_, which were reversed by NAC treatment in mock cells (Figure 2C, upper left panel). Induction of Twist expression, a metastasis-promoting transcription factor, was observed by H_2_O_2_ treatment in mock cells (Figure 2C, lower left panel). On the other hand, in SOCS1 over-expressing cells, the expression of vimentin and Twist was notably reduced while E-cadherin levels elevated (Figure 2C, right panels). These data strongly suggest that ROS signal induces EMT and that SOCS1 has an anti-EMT effect in colon cancer cells.

**Figure 3:**
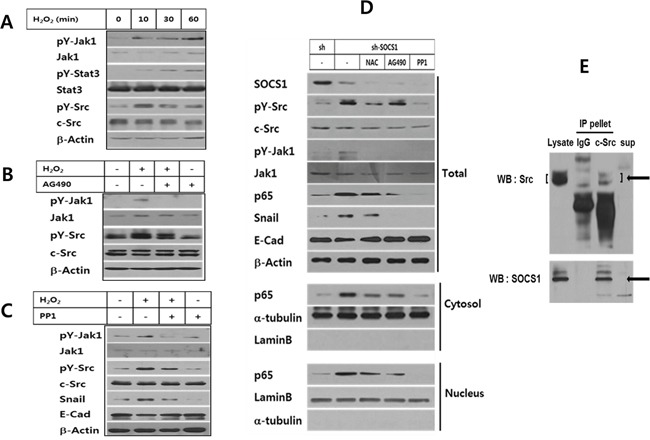
ROS-induced Jak and Src participate in EMT signaling, which are regulated by SOCS1 HCT116 p53 +/+ cells were treated with H_2_O_2_ at 200 μM for different durations and analyzed for Jak and Src activation by Western blot **A.** HCT116 p53 +/+ cells were treated with H_2_O_2_ at 200 μM for 2 h with or without pretreatment of JAK-inhibitor AG490 **B.**, or Src-inhibitor PP1 **C.** and changes in EMT markers and kinases were analyzed. HCT116 p53 +/+ sh & sh-SOCS1 cells were incubated in the absence or presence NAC, AG490 and PP1. Changes in EMT associated proteins or kinase were analyzed by Western blot **D.** To examine the molecular interaction of Src and SOCS1, HCT116 p53+/+ cells over-expressing SOCS1 were subject to co-immunoprecipitation assay. Cell lysates were incubated with rabbit monoclonal c-Src Ab or control rabbit IgG followed by protein A/G-agarose beads. The immunoprecipitated pellets were separated from the supernatant, which were resolved on SDS-PAGE along with total lysates, and subjected to immunoblotting to reveal SOCS1 and Src protein bands **E.**

Next, in order to delineate the mechanism of SOCS1 inhibition on EMT, ROS-induced signaling pathways leading to EMT were first examined. Jak and Src are of particular interest, since Jak is a classical target of SOCS1 [21] and Src is a potential regulator of EMT [17, 18, 34]. Both Jak1 and Src activation was noted upon exposure to H_2_O_2_ from 10 to 60 min (Figure 3A). While Jak inhibitor AG490 slightly suppressed Src activation, Src inhibitor PP1 strongly blocked both Jak1 and Src activation as shown by reduced tyrosine phosphorylation together with decreased expression of downstream molecules for EMT including p65 and Snail (Figure 3B and 3C). In shSOCS1 cells elevated pY-Src and pY-Jak1 were noted, which were both subject to a complete inhibition by PP1. Only a partial inhibition of pY-Src was obtained by AG490 treatment. Still both AG490 and NAC suppressed shSOCS1-induced increase of Snail and nuclear p65, while restoring E-cadherin levels. The result suggests that shSOCS1-induced promotion of EMT signal involves Jak and Src activated by ROS (Figure 3D). SOCS1 inhibition of Jak is known to involve molecular interaction of SOCS1 with tyrosine phosphorylated Jak [33, 35]. SOCS1 also has been reported to bind other phosphorylated substrates, leading to SOCS-box mediated degradation [36, 37]. Hence, in order to find out whether the observed SOCS1 inhibition of Src involves interaction of the two molecules, co-immunoprecipitation assay was performed. In HCT116 cells transduced with SOCS1, the c-Src antibody immunoprecipitates contained SOCS1 along with Src (Figure 3E). The result suggests that SOCS1 binds with Src, which may serve as a mechanism of the SOCS1 inhibition of Src and the subsequent EMT signaling.

### Anti-EMT function of SOCS1 through ROS down-regulation is mediated by thioredoxin

**Figure 4:**
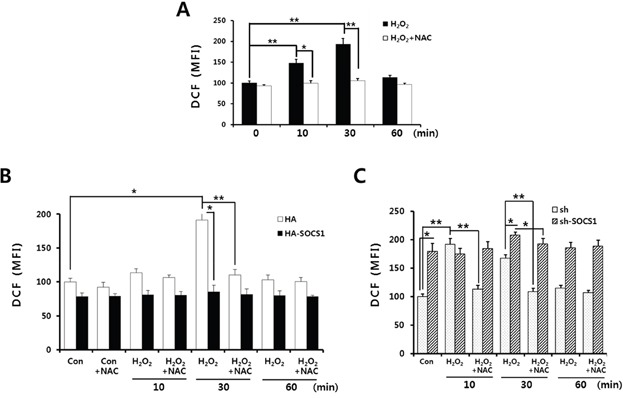
ROS suppressive function of SOCS1 as revealed by analysis of intracellular ROS levels upon SOCS1 or shSOCS1 transduction Cells were stimulated with H_2_O_2_ in the absence or presence of NAC pretreatment for 1 h. H_2_O_2_ treatments were done for indicated times and analysis of intracellular ROS levels was performed for parental HCT116 p53 +/+ **A.**, HCT116 p53 +/+ HA & HA-SOCS1 **B.**, and sh & sh-SOCS1 **C.**

**Figure 5:**
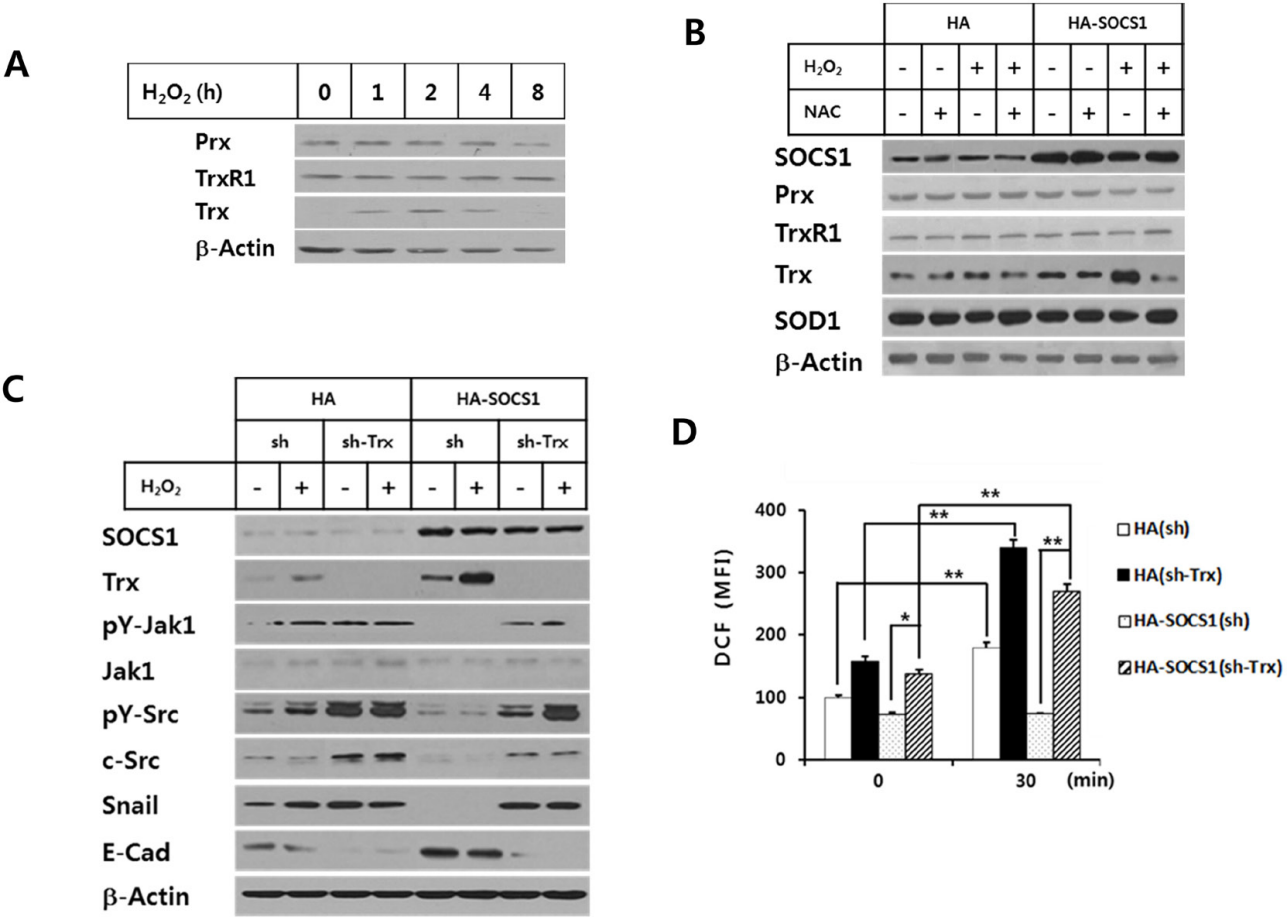
Role of thioredoxin induction by SOCS1 in down-regulation of ROS-mediated EMT signaling HCT116 p53 +/+ cells **A.** and HCT116 p53 +/+ HA & HA-SOCS1 cells **B.** were treated with H_2_O_2_ for different durations and antioxidant proteins were analyzed by Western blot. HA & HA-SOCS1 cells were each transfected with shRNA or shTrx and then treated by H_2_O_2_, after which changes in EMT markers and kinases were analyzed by Western blot at 2 h **C.** Analysis of intracellular ROS levels was performed for cells in Panel (C) at 30 min after H_2_O_2_ treatment **D.**

To identify the role of intracellular ROS signal during the hydrogen peroxide-induced EMT in HCT116 colon cancer cells, kinetics of changes in intracellular ROS levels were analyzed. Upon H_2_O_2_ treatment, ROS generation was detected from 10 min and peaked at 30 min which is decreased thereafter. ROS scavenging drugs NAC as well as DPI abrogated the ROS levels induced by hydrogen peroxide (Figure 4A and Supplementary Figure S1). Since cells transduced with SOCS1 exhibited reduced expression of EMT markers resembling the effect of anti-oxidant, we have examined the effect of SOCS1 or shSOCS1 on the intracellular ROS levels. HA and sh mock cells responded to hydrogen peroxide with a peak of ROS in 10 to 30 min, in a similar manner to the parental HCT 116 cells. However, intracellular ROS levels were kept significantly low in SOCS1-transduced cells, whereas substantially high in SOCS1-ablated cells (Figure 4B and Figure 4C). Both cells are resistant to changes in ROS levels upon extrinsic oxidant stimulation, which correlates with rather consistent expression of EMT markers. They are observed at low levels upon SOCS1 over-expression and high levels upon SOCS1 knock-down independent of H_2_O_2_ treatment (Figure 2B). To investigate the mechanism by which ROS dysregulation occurs by SOCS1, the induction of anti-oxidant enzymes was analyzed. As shown in Figure 5A, hydrogen peroxide treatment at concentrations used for EMT induction leads to transient up-regulation of thioredoxin (Trx1) from 1 to 4 h, but not of the other anti-oxidant enzymes, such as peroxiredoxin (Prx), thioredoxin reductase (TrxR1), and superoxide dismutase (SOD1). In SOCS1 over-expressing cells, thioredoxin level is up-regulated both at basal and H_2_O_2_-induced conditions (Figure 5B).

To find out whether the elevated thioredoxin is responsible for the reduced ROS level and the subsequent inhibition of EMT response upon SOCS1 transduction, we have examined the effect of thioredoxin ablation by shTrx transduction into HA-SOCS1 cells. The result demonstrates that shTrx transduction abrogated the SOCS1-induced anti-EMT effect, as shown by restoration of Snail expression with suppression of E-cadherin levels (Figure 5C). Such reversal in EMT response was correlated well with restored ROS levels in HA-SOCS1 cells upon shTrx transduction (Figure 5D). The ROS-induced activations of Src and Jak were also recovered by thioredoxin ablation in HA-SOCS1 cells (Figure 5C). Together these data strongly suggest that SOCS1-induced ROS down-regulation and EMT inhibition occur through thioredoxin up-regulation.

### Role of Src inhibition in thioredoxin up-regulation by SOCS1

**Figure 6:**
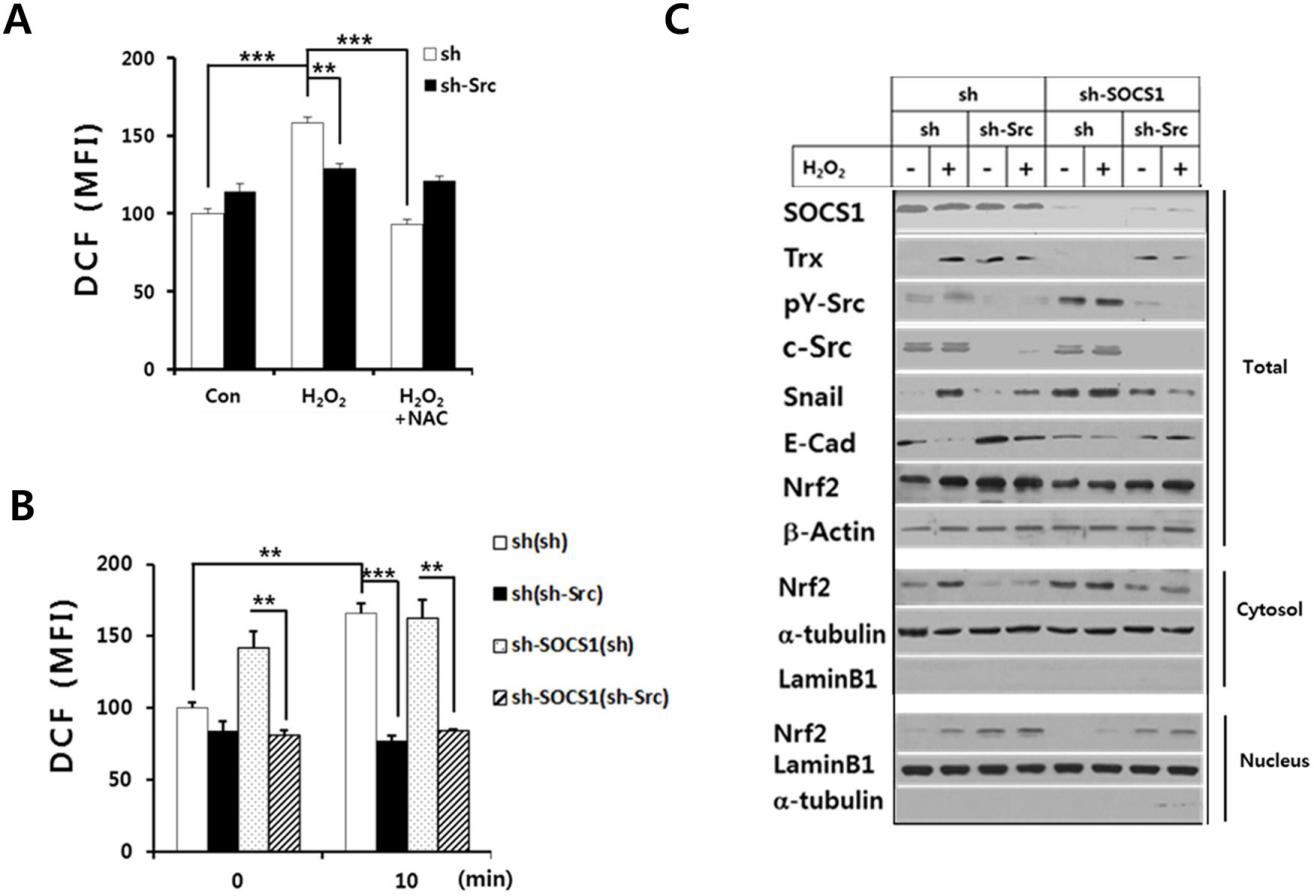
Role of Src in the H_2_O_2_-induced intracellular ROS and thioredoxin/Nrf-2 regulation by SOCS1 HCT116 p53 +/+ cells were transfected with sh or shSrc construct as described in the text and cells were analyzed for ROS levels upon treatment with H_2_O_2_ at 30 min with or without NAC pretreament **A.** sh and shSOCS1 cells transfected with sh or shSrc were subject to treatment with H_2_O_2_. The intracellular ROS levels were determined at 30 min **B.** Cells in panel B were treated with H_2_O_2_ for 2 h, harvested to obtain subcellular fractions, and analyzed for the expression of thioredoxin (Trx1), Nrf-2, and EMT markers by immunoblotting **C.**

In order to elucidate the mechanisms for the reciprocal regulation of thioredoxin and ROS by SOCS1 to attenuate EMT in colon cancer cells, we have investigated the function of Src as a mediator of SOCS1 action. Src has been implicated in both ROS production and regulation of antioxidant factors [19, 38, 39]. In particular, Src is shown to participate in ROS generation through the activation of the Nox system [19]. On the other hand, Src family enzyme-induced tyrosine phosphorylation of Nrf-2 is shown to be required for its nuclear export, which serves as a mechanism inhibiting Nrf-2 activity, an anti-oxidant transcription factor [38]. Thus, the effect of Src ablation on the H_2_O_2_-induced intracellular ROS was examined. As shown in Figure 6A, shSrc transduction significantly reduced intracellular ROS levels induced upon H_2_O_2_ stimulation. More importantly, while shSOCS1 cells displayed enhanced ROS levels at both basal and oxidant-treated conditions, Src ablation negated the shSOCS1-induced up-regulation of ROS (Figure 6B). In correlation with this, elevation of thioredoxin expression was evident upon shSrc transduction. It should be noted that shSOCS1 caused reduced thioredoxin level with enhanced Src activity and EMT markers, which were all reversed by Src ablation with shSrc (Figure 6C). In mock (sh) cells, shSrc transduction up-regulated both total and nuclear Nrf-2 while reducing cytosolic Nrf-2. In shSOCS1 cells such phenomena were reversed which were in turn, mostly recovered by shSrc: shSOCS1-induced decrease in nuclear Nrf-2 is restored by shSrc to correlate with the substantial levels of thioredoxin expression (Figure 6C).

**Figure 7:**
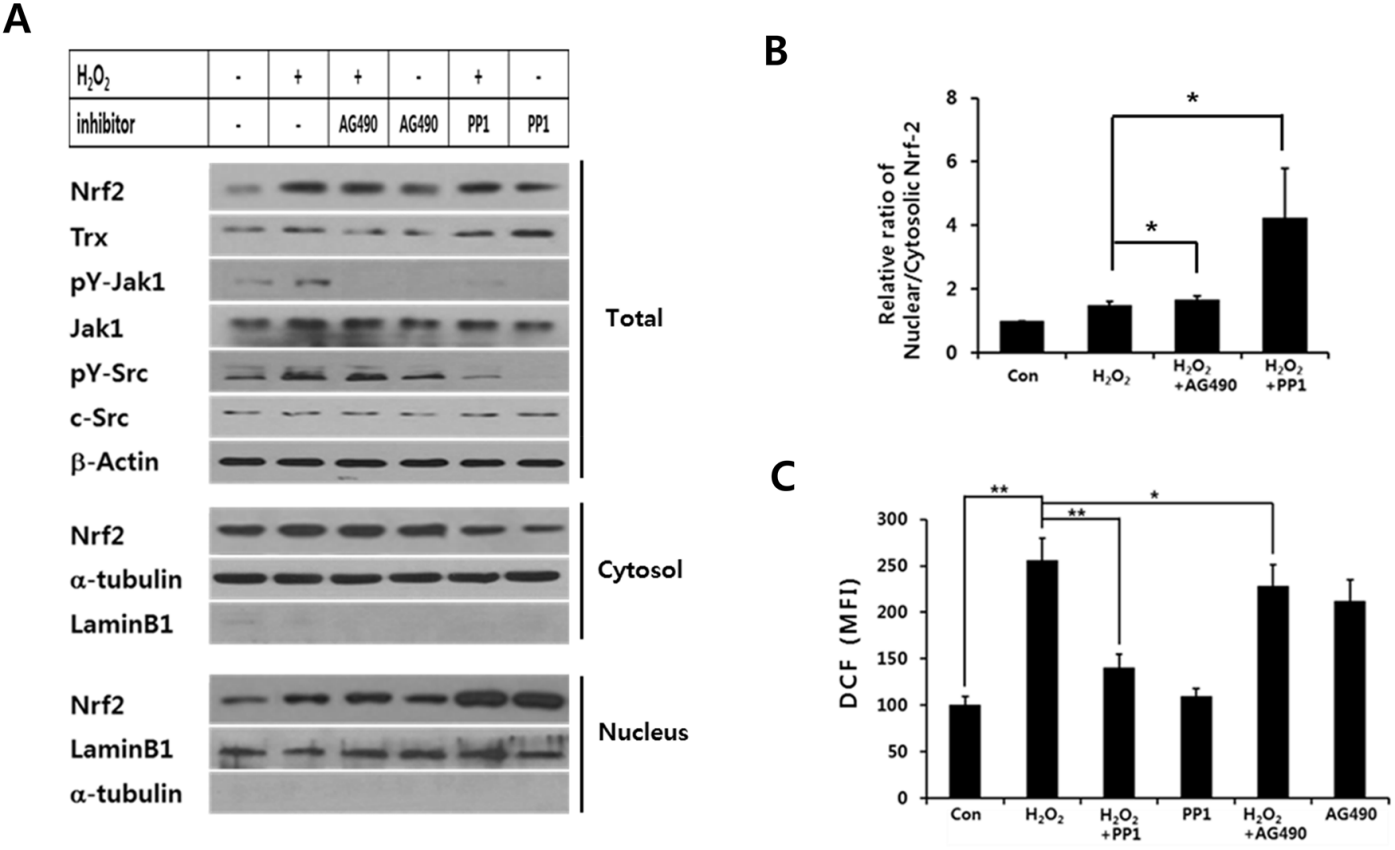
Src inhibitor but not Jak inhibitor induces nuclear retention of Nrf-2 and suppression of intracellular ROS levels HCT116 p53 +/+ cells were stimulated with H_2_O_2_ at 200 μM for 2 h with or without PP1 or AG490 treatment. Cell lysates were analyzed for Src, Jak, and Trx. Nrf-2 levels were further analyzed in total, cytosolic and nuclear preparations **A.** Nuclear to cytosolic ratio of Nrf-2 was determined from densitometry analysis of blots with cells treated with PP1 or AG490 prior to H_2_O_2_ stimulation **B.** Intracellular ROS levels were measured at 30 min post H_2_O_2_ stimulation **C.** Data in (B) and (C) represent mean+SE from 3 independent sets of experiments.

The data demonstrate that the increase of ROS levels caused by shSOCS1 is dependent on Src. To confirm the function of Src activity in nuclear vs cytosolic retention of Nrf-2, the effect of PP1 was analyzed (Figure 7). Similar to the case of Src knock-down, the inhibition of Src activity by PP1 resulted in up-regulation of nuclear Nrf-2 at the expense of decrease in cytosolic Nrf-2 (Figure 7A, lanes 5 and 6). PP1-treated cells exhibited increased thioredoxin expression with a significant reduction of intracellular ROS levels upon H_2_O_2_ treatment (Figure 7A and 7C). As compared to PP1, Jak inhibitor AG490 has little effects on the suppression of ROS level and Nrf-2 translocation under H_2_O_2_ treatment. Nuclear to cytosolic ratio of Nrf-2 was notably increased by PP1 but not by AG490 (Figure 7B). Together the data suggest that Src plays a negative role in Nrf-2-mediated anti-oxidant response such as induction of thioredoxin. Although Jak is an important target of SOCS1, inhibition of Src activity by SOCS1 appears primarily responsible for nuclear retention of Nrf-2 leading to thioredoxin expression. The enhanced ROS levels and pro-EMT signaling observed in shSOCS1 cell thus likely to be resulted from increased Src activity which induces down-regulation of thioredoxin.

### Anti-invasive effect of SOCS1 through ROS regulation

**Figure 8:**
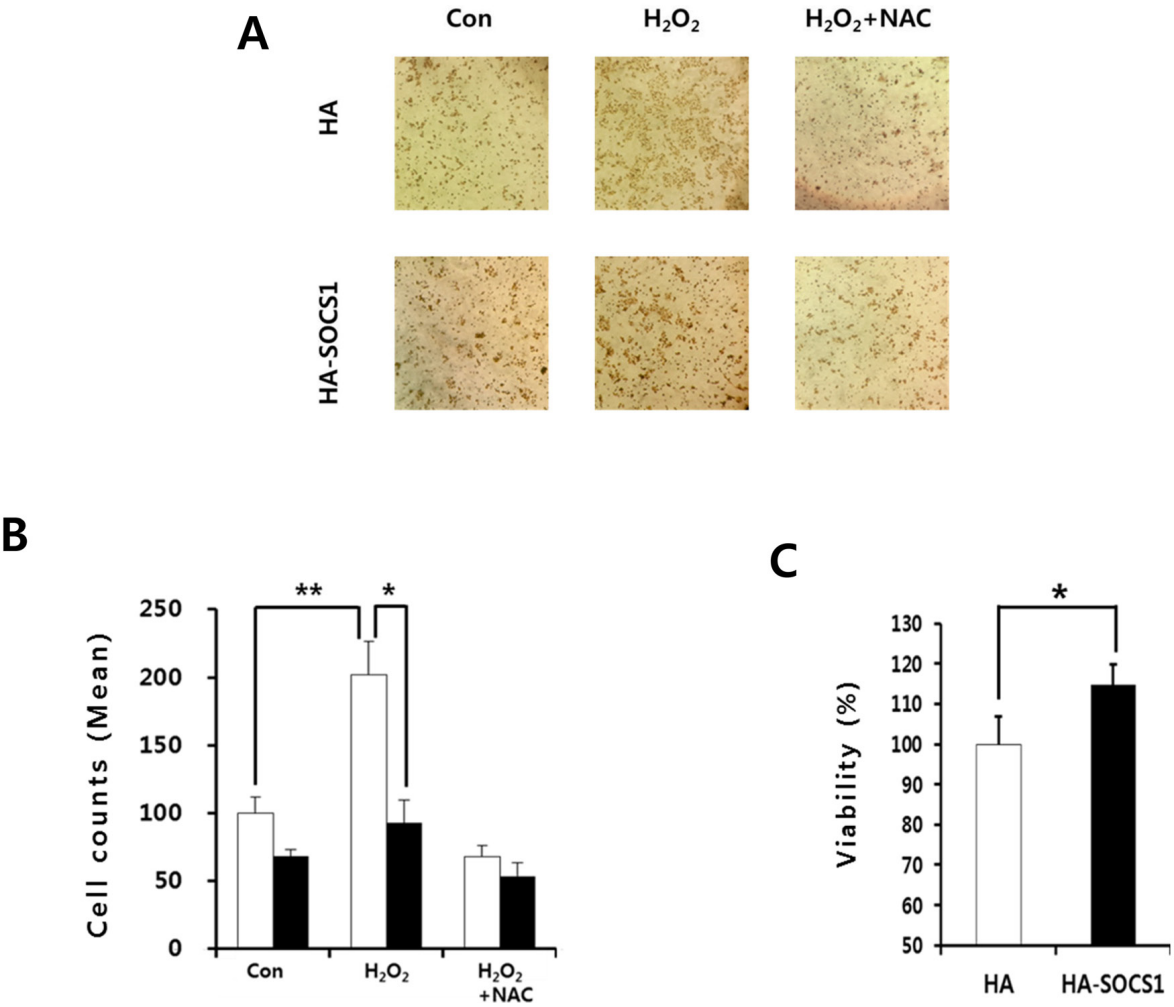
Inhibitory effects of SOCS1 on the H_2_O_2_-mediated invasiveness of the HCT116 colon cancer cells HCT116 HA & HA-SOCS1 cells were treated with H_2_O_2_ in the presence or absence of NAC pretreatment and the invasive properties were assessed by Matrigel assays as described in the text. Cells were stained with Hematoxylin **A.** and counted cell numbers are shown as mean+SE in bar graphs **B.** HCT116 HA & HA-SOCS1 cells were grown for 72 h and then examined for viability using MTT assays **C.**

The above data indicated that SOCS1 may prevent EMT signaling and response by activating the cellular anti-oxidant system. Because multiple factors involved in EMT are correlated with invasive property of tumor cells, we have examined the effect of SOCS1 in invasion capacity of HCT116 cells and the role of ROS in this process. The invasion assay was performed using a Matrigel chamber for mock and SOCS1-transduced cells. The hydrogen peroxide treatment shown to induce EMT increased the number of cells migrated through the Matrigel, which was inhibited by an anti-oxidant NAC. SOCS1-transduced cells exhibited a significantly reduced invasive capacity as compared to mock cells (Figure 8A and 8B). This is not due to the difference in cell growth, as no inhibition was observed for cell proliferation upon SOCS1 over-expression during the 72 h assay (Figure 8C). The low invasive properties of SOCS1-tansduced cells correlated with low ROS levels observed in these cells (Figure 4), suggesting that SOCS1 exhibits anti-invasive effect through down-regulation of ROS.

## DISCUSSION

Aberrant activation and discordant regulation of Jak/STAT and SOCS pathways have been associated with different malignancy for colon cancer specimens [30, 31, 40]. Considering the potential effect of ROS generated during the therapy-induced response on cancer progression, we have investigated the anti-EMT function of SOCS1 through ROS regulation in colon cancer cells transduced with SOCS1 and shSOCS1.

Regarding the anti-EMT action of SOCS1, David et al. [41] have recently reported that SOCS1 modulates invasion and metastatic potential of SW620 colon cancer cells through up-regulation of the miRNA200 family. In this report, upon over-expression of SOCS1, SW620 cells exhibited reversal of EMT features including re-expression of E-cadherin and suppression of ZEB1. While the study suggested anti-invasive function of SOCS1 is through the induction of miRNA200 known to down-regulate ZEB1, the mechanism by which SOCS1 induces miRNA200 remains unknown leaving the upstream signaling pathways of SOCS1 action for investigation. In addition, while they have utilized highly aggressive colon cancer cells with low expression of SOCS1 for SOCS1 transduction, the effect of SOCS1 ablation was not presented.

We have chosen colon cancer cells with SOCS1 expression at intermediate levels through initial screening of various colorectal cancer cell lines (data not shown). HCT116 cells were selected since they are amenable for both SOCS1 over-expression and knock-down. Our data demonstrate that while ROS stimulation can induce EMT features in these cells, SOCS1 vs shSOCS1 transfection produces opposite features on EMT regulators which are correlated with changes in intracellular ROS levels (Figures 1, 2, 4). Furthermore the suppression of EMT signaling by SOCS1 was consistent with its blocking effect on the ROS-induced invasion as revealed by Matrigel assays (Figure 8).

The mechanism by which SOCS1 down-regulates the intracellular ROS levels was then investigated, which demonstrated that thioredoxin up-regulation by SOCS1 is a primary mode. There were no apparent changes in other anti-oxidant enzymes, such as superoxide dismutase, peroxiredoxin or thioredoxin reductase in SOCS1-transduced cells. More importantly thioredoxin depletion abolished SOCS1-induced suppression of ROS, Src, Jak, p65 activity and EMT markers, suggesting that anti-ROS and anti-EMT functions of SOCS1 are mediated through thioredoxin up-regulation (Figure 5). Such anti-ROS action of SOCS1 is in line with our previous study, in which ROS-mediated apoptosis of Jurkat T cells by TNF-alpha or hydrogen peroxide is inhibited by SOCS1 through thioredoxin induction [33].

In the present work we have further examined the mechanism of thioredoxin regulation by SOCS1 and obtained data indicating that inhibition of Src activity is responsible for the promotion of thioredoxin expression through nuclear retention of Nrf-2. Nrf-2 is a transcription factor critical for the induction of anti-oxidant enzymes by binding to the anti-oxidant response element of promoters for HO-1, SOD1 and thioredoxin [39, 42, 43]. The finding that Src family kinase Fyn activity-dependent phosphorylation of Nrf-2 is required for nuclear export [38, 39] provides an explanation for the increase in nuclear Nrf-2 levels upon Src inhibition (Figure 7A). As Src activity is reciprocally regulated by SOCS1 and shSOCS1, increased Src activity in shSOCS1 cells can be responsible for decreased nuclear Nrf-2 levels as compared to mock cells (Figure 6C).

Although both Jak and Src are regulated by SOCS1, Src appears to act upstream of Jak and to be a dominant target of SOCS1 to down-regulate ROS signal in colon cancer cells. PP1 but not AG490 promoted nuclear retention of Nrf-2 with thioredoxin induction and suppressed intracellular ROS levels upon H_2_O_2_ treatment (Figure 7). It should be noted that among various anti-oxidant factors, thioredoxin is a primary enzyme up-regulated upon SOCS1 over-expression and Src ablation (Figure 5B and Supplementary Figure S2). Thioredoxin is also shown to bind and protect protein tyrosine phosphatases (PTP) from the ROS-induced inactivation thus attenuating Jak activity [33, 44]. Hence thioredoxin up-regulated in SOCS1-transduced HCT116 cells, may further inhibit Src and Jak by maintaining PTP activities.

Tumor cells often exhibit high intracellular ROS levels due to increased metabolism and adapt to the oxidative stress caused by exogenous ROS produced in inflammatory tumor environment [45]. In addition, increased ROS production by Nox system activated by oncogenic pathways such as Src [19] would also contribute to EMT. In this regard, SOCS1 may inhibit ROS-mediated EMT process by down-regulation of ROS as well as Src activity. The molecular interaction noted between Src and SOCS1 suggest a possible mode of Src kinase inhibition by SOCS1 (Figure 3E). While SOCS1 is known to cause proteolytic degradation of tyrosine-phosphorylated substrates upon binding, the degradation of c-Src by SOCS1 was not noted in HA-SOCS1 cells (Supplementary Figure S3). Thus, the activity regulation rather than degradation of Src appears responsible for the attenuation of downstream signaling for EMT. In addition to the decreased Src activity, the SOCS-box mediated degradation of signaling molecules such as NF-κB/p65 may also aid the anti-EMT function of SOCS1 [32, 46].

**Figure 9:**
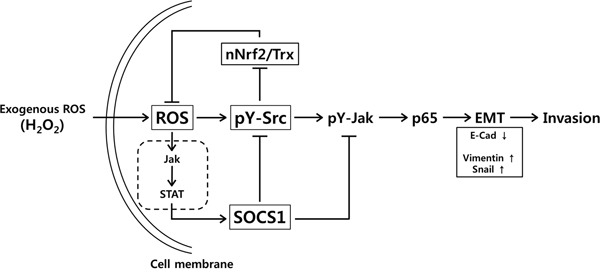
Model for the mechanism of SOCS1 action in inhibition of EMT signaling through ROS suppression involving Src-mediated thioredoxin regulation in colon cancer cells In HCT116 cells, intracellular ROS generated upon exogenous hydrogen peroxide treatment stimulates Src and Jak kinase activation leading to p65 activation and modulation of EMT regulators such as up-regulation of vimentin and Snail accompanied with down-regulation of E-Cadherin. SOCS1 induced by ROS signal suppresses Src activity involving molecular interaction. The pY-Src activity is responsible for attenuation of nuclear Nrf-2 (nNrf-2) and thioredoxin (Trx) levels. Src inhibition by SOCS1, thus results in increased Trx and decreased ROS levels in SOCS1-transduced cells. Src inactivation leads to the reduced EMT signaling and invasion property. Jak inhibition during this process can be achieved directly by SOCS1 and via suppression of Src by SOCS1.

In summary, we have shown that ROS signal potentially induces EMT in colon cancer cells through Src activation and that SOCS1-induced inhibition of Src activity leads to the up-regulation of thioredoxin via Nrf-2, thereby inhibiting EMT. Down-regulation of ROS by SOCS1 can be resulted from both the inhibition of Src-mediated Nox activity [19] and the promotion of thioredoxin-mediated ROS scavenging activities [47]. Thus SOCS1 is likely to participate in the ROS regulatory loop involving Src and thioredoxin (summarized in Figure 9). By reducing intracellular ROS found at abnormally high levels in cancer cells, SOCS1 may exert anti-EMT and anti-invasive functions to prevent malignant progression. Such tumor suppressive functions of SOCS1 through the activation of cellular anti-oxidant defense system would be particularly useful in control of colon cancers surviving high oxidative stress induced upon chemo- or radiation therapy.

## MATERIALS AND METHODS

### Antibodies

Anti-Snail, anti–E-Cadherin, anti–N-Cadherin, anti–Slug, anti–peroxiredoxin(Prx), anti-thioredoxin(Trx1), anti-phosphotyrosine(Y416)-Src, anti–phospho-Erk1/2, anti-phosphotyrosine-Jak1, anti-phosphotyrosine-Stat1, and anti-tubulin Abs were from Cell Signaling Technology (Beverley, MA). Anti-SOCS1, anti-vimentin1, anti-TrxR1, and anti–SOD1 Abs were purchased from Santa Cruz Biotechnology (Santa Cruz, CA). Anti-p65 Ab was from Upstate Biochemicals (Charlottesville, VA). Anti-LaminB1 and anti-c-Src Abs were from Abcam (Cambridge, MA).

### Cell culture

Human colon cancer cell lines HCT116 p53 +/+ (p53 wt), HCT116 p53 −/− (p53 null) and HT29 (p53 mt) were obtained from the American Type Culture Collection. Cells were cultured in complete DMEM supplemented with 10% FBS (Invitrogen, Carlsbad, CA), 10 mM HEPES, 2 mM L-glutamine, 50 μM 2-ME, 10,000 U/ml penicillin, and 10,000 μg/ml streptomycin (Life Technologies, Grand Island, NY) at 37°C under 5% CO_2_. HA & HA-SOCS1/HCT116 p53 +/+ cells were cultured and maintained with 200 μg/ml G418 (Sigma-Aldrich). Sh & sh-SOCS1/HCT116 p53 +/+ cells were maintained with 100 ng/ml puromycin (Sigma-Aldrich). shSrc-transfected sh-SOCS1/HCT116 or shTrx-transfected HA-SOCS1 cells were maintained with puromycin or G418 plus puromycin, respectively. Cells were cultured in a humidified 5% CO_2_ incubator at 37°C.

### Hydrogen peroxide and inhibitor treatments

Cells were treated with hydrogen peroxide (H_2_O_2_, Mpbio) at different doses and durations. For optimal induction of EMT markers, the parental HCT116 cells were treated with 200 μM H_2_O_2_, whereas HA or sh vector-transfected cells were treated with 300 μM H_2_O_2_, unless otherwise indicated. Treatment with inhibitors (NAC, AG490, and PP1: Sigma Aldrich) was done prior to cellular stimulation with hydrogen peroxide. NAC and AG490 were treated at 10 mM and 100 μM for 1 h, respectively. PP1 was at 5 μM for 4 h.

### Gene transfection

HA & HA-SOCS1, sh & sh-SOCS1, and shTrx1 constructs were previously described [33]. The shSrc constructs were obtained from Sigma Aldrich (NM 198291.1 CCGGGTCATGAAGAAGCTGAGGCATCTCGAGATGCCTCAGCTTCTTCATGACTTTTTG). For transfection, HCT116 cells in 500 μl Opti-MEM buffer (Invitrogen) were mixed with 5 μg each of pcDNA-HA, HA-SOCS1, shRNA vectors and shRNA against SOCS1, Src and Trx. The cell mixture was transferred into a 4 mm electrode gap cuvette and transfection was performed using Gene Pulser X cell electroporation system (Bio-Rad, Herbercules, CA).

### Immunofluorescence analysis

Fix-permeabilized cells were stained with primary antibodies (E-Cadherin, Twist1, vimentin, and SOCS1), followed by incubation with fluorescence-conjugated secondary antibodies (Alexa-488, Alexa-594: Molecular probe, Eugene, OR, USA and TRITC: Biofixs, Tampere, Finland). Nuclear staining was performed with Hoechst 33342 (Molecular probe). After extensive washing, cells were analyzed by using a confocal microscope (LSM 710 Meta DuoScan, Carl Zeiss Micro Imaging GmbH, Germany) equipped with a 40X objective.

### MTT assays

Cell viability was determined with a colorimetric MTT assay using 3-[4,5-Dimethylthiazol-2-y]-2,5-diphenyltetrazolium bromide (Promega).

### Analysis of intracellular ROS levels by FACS

Cells were stained with 1 μM H_2_DCF-DA 20 min before the end of the incubation with hydrogen peroxide. Fluorescence was measured with excitation at 480 nm and emission at 530 nm to assess intracellular ROS levels using the FACS Calibur flow cytometry system equipped with CELLQuestpro software (BD Bioscience).

### *In vitro* invasion assay

An *in vitro* invasion assay was performed using a Matrigel kit (Chemicon), according to the manufacturer's protocol. Invasiveness was evaluated by staining cells that had migrated through the extracellular matrix layer and adhered to the polycarbonate membrane at the bottom of the insert during the 48 to 72 h assays. Numbers of cells adhering to different regions of the bottom of the insert were counted at 200X magnification.

### Western blot analysis and co-immunoprecipitation assay

Whole cell, cytosolic, or nuclear lysates were isolated as previously described [32]. The lysates (60 μg each) were separated on SDS-PAGE and transferred to polyvinylidene difluoride membranes. After incubation with primary Abs, the immunoblots were revealed by HRP-conjugated anti-mouse, anti-rabbit or anti-rat secondary Abs (Cell Signaling Technologies and Santa Cruz) and detection with Immobilon Western (Millipore, Billerica, MA). For immunoprecipitation, cell extracts were prepared in immunoprecipitation buffer (10 mM HEPES [pH 7.6], 15 mM KCl, 2 mM MgCl2, 0.1% Nonidet P-40, 1 mM PMSF) and complete protease inhibitor (Roche). The extracts (600 μg proteins) were incubated with rabbit monoclonal anti-Src Ab(Abcam) or rabbit IgG for 12 h at 4°C. Protein A/G-agarose beads (Santa Cruz Biotechnology) were then added, after which the bound proteins were released, resolved on SDS-PAGE, and analyzed by immunoblotting.

### Densitometry analysis

The densitometric analysis of immunoblots was performed using ImageJ software.

### Statistical analysis

All experiments were performed at least in three independent sets. The values are presented as means ± SE. Statistical significance was determined by a Student-t-test. A value of * p < 0.05, ** p < 0.01 and *** p < 0.001 was considered statistically significant.

## SUPPLEMENTARY FIGURES


