# Supplementary material for: Mechanism of suppressors of cytokine signaling 1 inhibition of epithelial-mesenchymal transition signaling through ROS regulation in colon cancer cells: suppression of Src leading to thioredoxin up-regulation
Source: Oncotarget. 2016 Aug 23;7(38):62559–71. doi: 10.18632/oncotarget.11537 (PMC5308746; doi:10.18632/oncotarget.11537)
Supplement: Supplementary file 1 [file oncotarget-07-62559-s001.pdf]

Mechanism of suppressors of cytokine signaling 1 inhibition of epithelial-mesenchymal transition signaling through ROS regulation in colon cancer cells: suppression of Src leading to thioredoxin up-regulation

SUPPLEMENTARY FIGURES

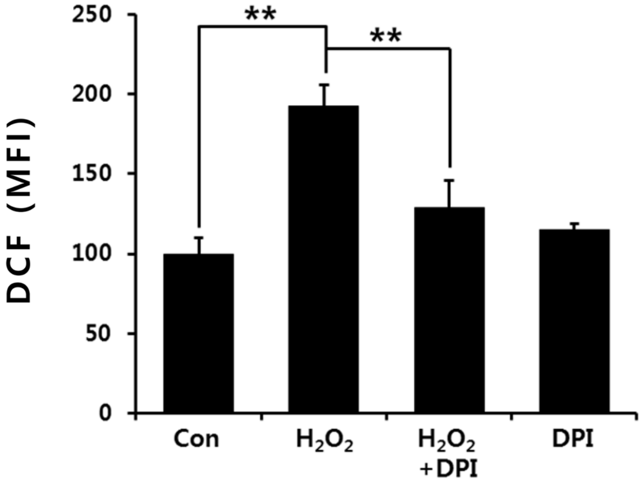

Supplementary Figure S1: Regulation of H_2O_2 -induced intracellular ROS levels upon treatment with DPI. HCT116 p53 +/+ cells were treated with 200 µM H_2O_2 in the absence or presence of DPI for 30 min and analysis of intracellular ROS levels was performed as described in the text.

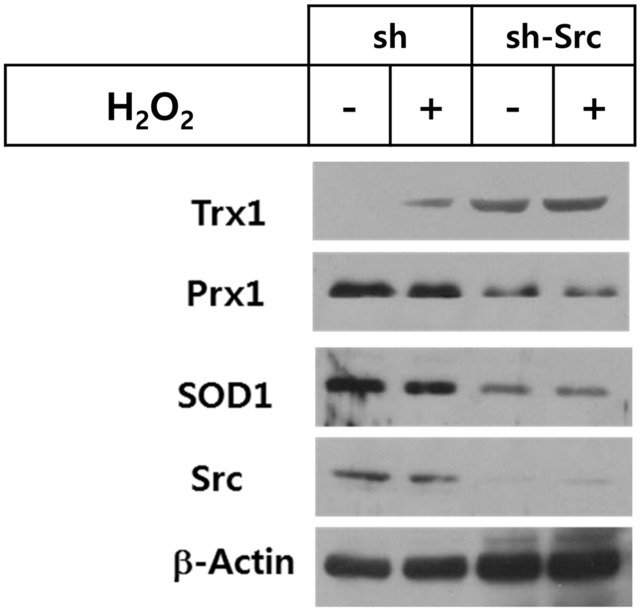

Supplementary Figure S2: Regulation of anti-oxidant factors by Src. HCT116 p53+/+ cells transfected with sh or shSrc were treated with H_2O_2 for 2 h and the expression levels of antioxidant factors were analyzed by immunoblotting.

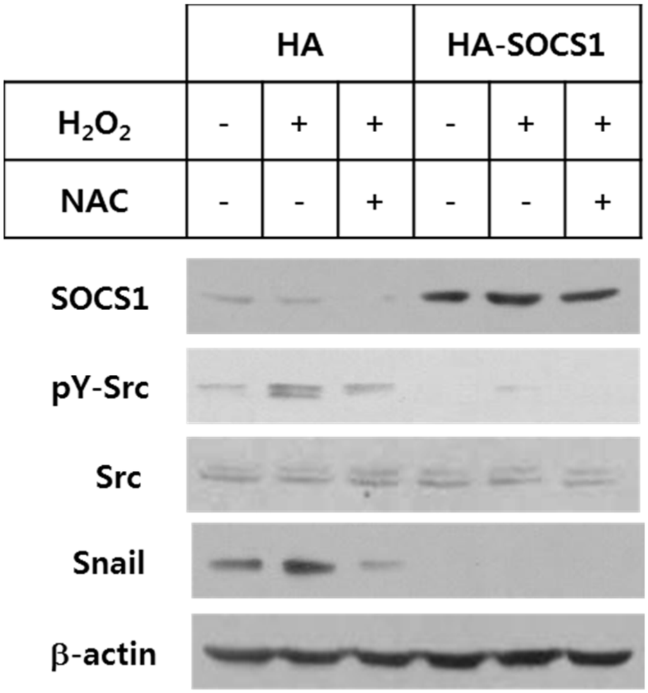

Supplementary Figure S3: SOCS1 over-expression down-regulates pY-Src levels without affecting Src levels in HCT116 cells. HCT116 p53 +/+ cells transduced with HA and HA-SOCS1 were treated with H_2O_2 and NAC. The expression levels of Src, pY-Src, and Snail were analyzed by immunoblotting.
